# Knowledge, Attitude, and Performance Among First High School Students in the Prevention of COVID-19 in Schools of Khorramabad City in 2022

**DOI:** 10.1155/ipid/8870159

**Published:** 2025-11-29

**Authors:** Salah Apsham, Ali Pajouhi, Soodabeh Zare, Amir Mohammad Karimi, Pegah Shakib, Azadeh Jafrasteh

**Affiliations:** ^1^Student Research Committee, Lorestan University of Medical Sciences, Khorramabad, Iran; ^2^USERN Office, Lorestan University of Medical Sciences, Khorramabad, Iran; ^3^Department of Biostatistics and Epidemiology, School of Health and Nutrition, Lorestan University of Medical Sciences, Khorramabad, Iran; ^4^Razi Herbal Medicines Research Center, Lorestan University of Medical Sciences, Khorramabad, Iran; ^5^Department of Pediatrics, School of Medicine, Lorestan University of Medical Sciences, Khorramabad, Iran

**Keywords:** attitude, awareness, COVID-19, knowledge, performance, students

## Abstract

**Background:**

The purpose of this study was to investigate the knowledge, attitude, and performance of the first secondary-level students in preventing COVID-19 in the schools of Khorramabad in 2022.

**Methods:**

In our study, the information, knowledge, attitude, and performance of 370 first-grade secondary school students were recorded using a standard questionnaire. Data were analyzed using SPSS Version 26 software and descriptive statistical methods (mean and standard deviation) and appropriate inferential tests to compare groups and examine the correlation between knowledge, attitude, and performance.

**Findings:**

The average score of knowledge, attitude, and performance was 59.11 ± 20.51, 81.25 ± 13.68, and 65.4 ± 23.1. The average score of knowledge in students over 14 years of age, ninth-grade students and good academic status, the score of knowledge and attitude of students with mothers with university education, and finally, the average score of knowledge, attitude, and performance of students with underlying disease were significantly higher than other students. Also, knowledge had a direct and significant relationship with attitude and performance. In addition, attitude and performance also had a direct and significant relationship.

**Conclusion:**

The level of knowledge of the people was average, and attitude and performance were positive. Based on the importance of performance, people's attitude towards COVID-19 should be improved, because knowledge alone cannot improve people's performance.

## 1. Introduction

Human history has always experienced various pandemics; tackling them is considered a serious concern. The 2019-2022 pandemic caused by severe acute respiratory syndrome coronavirus-2 (SARS-CoV-2) is estimated to cause over 18 million deaths [1, 2]. Moreover, possible long-term health complications of COVID-19 involve mental health and many human systems, including immune, hematological, pulmonary, cardiovascular, gastrointestinal, musculoskeletal, and nervous systems [3]. Given the risks posed by this crisis, preventive measures seem crucial. Initial preventive strategies were suggested for close contact avoidance, home-staying, frequent hand washing, and mask-wearing [4].

How civilians react to the preventive methods results in critical consequences. The predictors of adherence to health guidelines during the pandemic are the collaborative elements, including mutuality, trust norms, and knowledge of one's role [5]. Adolescents are considered a vulnerable demographic within society during the COVID-19 pandemic [6]. It seems that the poor performance in disease prevention by this group can significantly hinder managing the pandemic. Therefore, in this study, the status of adolescent students was examined in the three areas of knowledge, attitude, and practice (KAP) (the health behavior triad) in preventing virus transmission. The results of this study can assist in determining which groups of adolescents should be the focus of preventive education during pandemics.

## 2. Methods

### 2.1. Type of Study and Sample Size

In this descriptive cross-sectional study, the studied population included all first-year high school students studying in the academic year of 2022 in Khorramabad city.

According to similar studies [7], considering the correlation coefficient equal to 0.15 with the Type 1 error equal to 0.05 and the test power equal to 0.8, using the PASS sample size determination software, it was calculated as 346 students. Considering the 10% drop, the final sample size was 370 students. To select the samples, Khorramabad city was first divided into three parts: North, Central, and South, and each part were considered a cluster. Then, girls' and boys' schools were regarded as classes in each section. In each section, one boy's school and one girl's school were randomly selected. In each school, 7th- to 9th-grade students were chosen by a simple random method and entered the final sample if they wanted to participate in the study. Informed consent was obtained from the subjects, who were assured that their data would remain confidential.

In this study, written consent was not obtained from parents because the study only involved voluntary completion of an anonymous questionnaire without any intervention. Before distributing the questionnaire, the objectives of the research and confidentiality of the information were explained to the students, and their participation was completely voluntary.

The present study received ethics approval with code (IR.LUMS.REC.1402.082) from Lorestan University of Medical Sciences.

### 2.2. Data Collection

The questionnaire used in this study includes demographic questions, questions related to knowledge, 11 questions about knowing the cause of the disease, mode of transmission, methods of prevention and treatment, and the importance of home quarantine (such as coronavirus currently does not have a particular drug treatment), attitude 16 questions about coronavirus (like because my body is strong, I don't get corona) and performance 3 questions (like I haven't left the house except for essential things in the last few days). Knowledge, attitude, and performance questions were answered using a Likert scale. The scores of the knowledge questions are from zero to one, and attitude and performance are from one to three. For the ease of comparison of knowledge, attitude, and performance areas, the scores of these areas were equalized and reported. The content validity of the questionnaire made by the researcher was confirmed by 5 health education and health promotion experts, and the CVI and CVR values were calculated as 0.87 and 0.9, respectively. The reliability of this questionnaire was also obtained through Cronbach's alpha test of 0.9 [8].

### 2.3. Data Analysis

The obtained data were analyzed using SPSS Version 26 software. First, the data were checked for normality of distribution. For descriptive analysis, mean and standard deviation were reported for quantitative data and frequency and percentage for qualitative data. To examine the differences between groups in quantitative variables, independent *t*-test and one-way ANOVA were used. To compare qualitative variables, chi-square test was used. Also, the correlation between knowledge, attitude, and performance variables was examined using Pearson correlation coefficient. The significance level for all tests was set at 0.05.

## 3. Results

In this study, 370 first-year high school students were included in the study, of which 184 (49.7%) were boys and 186 (50.23%) were girls. The average age was 14.11 ± 0.91 years (13–17 years). The findings of the present study are shown in Table 1.

The average knowledge score was 59.11 ± 20.51, the average attitude score was 81.25 ± 13.68, and the average performance score was 65.4 ± 23.1 (Tables 1, 2, and 3). The average knowledge score of students over 14 years of age was significantly higher than that of students under 14 years of age, but no difference was observed in terms of attitude and performance (*p* > 0.05). The average knowledge and attitude score of students with mothers with university education was significantly higher than that of students with diploma education and below (*p* > 0.05), but no difference was observed in the performance score (*p* > 0.05). The average knowledge score was the highest in ninth-grade students (32.63) and the lowest in seventh-grade (13.55), which was a significant difference (*p*=0.011). However, no difference was observed in attitude and performance. The average knowledge score in students with good academic status was the highest (3.64) and the lowest (92.51) in students with poor academic status, which was a significant difference (*p* < 0.001). However, no difference was observed in attitude and performance. The average score of knowledge, attitude, and performance of students with underlying disease was significantly higher than that of students without underlying disease. There was a significant correlation between age and knowledge. It was also found that knowledge has a significant relationship with attitude and performance. Finally, it was found that attitude and performance also had a direct and significant relationship (Table 4).

## 4. Discussion

We concluded that age can be an important factor in the knowledge level of students, while gender has no impact on any aspect of student's health behavior. However, observations in Saudi Arabia, a country with geographic and cultural similarities to Iran, indicated that women possessed superior knowledge and exhibited better hand hygiene than men during the COVID-19 pandemic [9]. In this study, respondents had a positive attitude and acceptable performance in COVID-19 prevention. However, the level of knowledge in our study was moderate and was assessed to be slightly lower than that reported by Saudis. This difference may be attributed to several factors, such as differences in educational and health interventions, access to reliable information, and cultural and social differences.

In a study by Tajvar et al. in 2020, which examined the level of KAP of Iranian people regarding the use of masks to prevent and control the spread of COVID-19. Among participants who were over 15 years old from all over Iran, only 13.7% of participants had good knowledge about the use of masks. 74% of participants had a positive attitude towards the use of masks to prevent and control COVID-19. 70.8% of participants had good performance in using masks. Based on the results, it was found that the attitude and practice of Iranian people regarding the use of masks were at a desirable level, but the level of general knowledge in this field needs to be improved [10].

In a study by Peng et al. in 2025 in China, which aimed to investigate the KAP of undergraduate students in the face of COVID-19, it was conducted in 10 universities in Shaanxi Province. The results of the survey on 872 students with an average age of 17 to 25 years old voluntarily and online showed that the average scores of KAP were 4.12 out of 5, 8.54 out of 10, and 8.91 out of 10, respectively. Also, a positive and significant relationship was observed between attitude and practice (*r* = 0.319). Comparison of this study with our study showed that in both countries, positive attitudes and acceptable practice in preventing COVID-19 were observed. However, in the Chinese study, the level of knowledge was on average higher than in our study. This difference may be due to the differences in educational systems, information sources, and health policies between the two countries. Also, in the Chinese study, there were significant differences in KAP scores based on gender, field of study, and type of university, while in our study, no such differences were observed [11]. Among the investigated variables in students and their families, mothers' educational degrees significantly impacted students' preventive measures (knowledge and attitude), while fathers' education showed no significant effect. Many students' mothers were homemakers, and many fathers were laborers or freelancers. Although we concluded that the parents' occupations do not affect preventive measures, the influence of mothers' literacy on students' knowledge and attitude may be linked to how much time mothers, especially homemakers, spend with their progeny during the day.

In our study, the knowledge and attitude of students about COVID-19 were good and very encouraging because correct and sufficient knowledge increases the safety of oneself and others against this disease. In the study of Ahmed Ayed et al. in India, the knowledge rate was reported to be 90%. In the study of Ahmed Ayed et al. 2021, educational intervention was emphasized to improve the reported knowledge, attitude, and performance of secondary school students and to prepare students to acquire more information [12]. In the study of Majhi et al. 2021, in India, the knowledge rate was reported to be 90.4%. In the study of Majhi, it was pointed out that due to the implementation of the online survey, response bias cannot be ignored in the current study. On the other hand, since the surveys were taken from only one institution, they may be unrepresentative and prevent the generalization of the results to other medical institutions in the country and the general population [13]. In the study by Wen et al. 2020, in China, 7377 students participated in a questionnaire survey from primary, middle, and high schools from 2 to 13 March 2020. The overall COVID-19 knowledge rate in the study was 74.1%. Thus, most students in Beijing had a high level of knowledge and optimistic attitudes towards COVID-19. However, targeted interventions were reported to be particularly necessary for students with high-risk characteristics (male, rural, and high school students) [7].

In the study by Aynalem et al. 2021, in Ethiopia, the reason for the high level of knowledge of the study subjects about this disease was explained as follows: Due to the late reporting of COVID-19 in this country, the necessary time was provided for the community to learn about this disease. The announcement of the World Health Organization, which described COVID-19 as a pandemic with high transmissibility, may have increased students' knowledge [14]. This is also true in our study. In a study by Noreen et al. 2020, KAP regarding COVID-19 among Pakistani medical students was examined. The survey results among 1474 medical students showed that Pakistani medical students had adequate attitudes towards COVID-19 and most recognized COVID-19 [15]. The difference in attitude between our studies with the mentioned study is probably due to the difference in the study population; in Noreen's study, the study population was medical students, while in the present study, the assessed population consisted of students. Thus, our study population was much younger and had a lower level of education than other studies; this may have caused the difference in the attitude score.

In the study by Azlan et al. 2020, the average knowledge score of Malaysians regarding COVID-19 was 10.5 ± 1.4 with an overall correct rate of 80.5%. However, the correct rates of COVID-19 knowledge varied widely, indicating that while some participants had high knowledge about the disease, others did not. Malaysians over 50 years of age had higher knowledge scores, possibly due to a higher perceived risk of complications. Also, those with lower monthly incomes had the lowest knowledge scores. This may indicate limited access to reliable and timely information about the virus. It was therefore suggested that this variation in knowledge levels may reflect the landscape of COVID-19 information in Malaysia [16].

## 5. Conclusion

The results of this study showed that first-year high school students had moderate knowledge, positive attitudes, and acceptable performance in COVID-19 prevention. The findings also indicated a positive and significant relationship between knowledge, attitude, and performance, indicating that increasing knowledge alone is not enough and that appropriate attitudes play an important role in improving preventive performance. In addition, higher maternal education was associated with increased knowledge and attitude of students, indicating that family factors can influence adolescents' knowledge and attitudes. These results can be used as a basis for designing targeted educational and promotional interventions to improve adolescents' preventive behaviors against infectious diseases.

## Figures and Tables

**Table 1:** The knowledge of first-year high school students in the prevention of COVID-19.

		*N*	Mean	SD	*p* value
Age	14 >	101	55.36	21.822	0.039
	14 ≥	269	60.53	19.856	

Gender	Boy	184	60.23	19.304	0.3
	Girl	186	58.02	21.636	

Mothers' education	Diploma or less	237	56.77	20.921	0.003
	University degree	133	63.29	19.135	

Fathers' education	Diploma or less	249	58.27	20.832	0.248
	University degree	121	60.86	19.808	

Mother's job	Housewife	309	59.31	20.558	0.679
	Employed	61	58.12	20.414	

Father's job	Unemployed	10	58.18	24.693	0.754
	Worker	104	60.05	21.326	
	Employee	38	60.05	23.870	
	Freelance	191	57.92	18.591	
	Other	27	62.96	24.305	

School level	7th	108	55.13	21.822	0.011
	8th	147	58.75	17.908	
	9th	115	63.32	21.705	

Educational status	Bad	90	51.92	25.938	< 0.001
	Average	211	60.49	17.131	
	Good	69	64.30	19.863	

Child's underlying condition	Negative	350	58.47	20.472	0.009
	Positive	20	70.45	18.152	

Contracting COVID-19	Negative	19	60.29	14.599	0.73
	Positive	351	59.05	20.797	

Total	Min: 0	370	59.11	20.511	
	Max: 100.00				

**Table 2:** The attitude of first-year high school students in the prevention of COVID-19.

		*N*	Mean	SD	*p* value
Age	14 >	101	81.90	13.494	0.573
	14 ≥	269	81.01	13.778	

Gender	Boy	184	80.55	13.956	0.331
	Girl	186	81.94	13.420	

Mothers' education	Diploma or less	237	79.97	14.591	0.011
	University degree	133	83.53	11.617	

Fathers' education	Diploma or less	249	80.84	14.052	0.391
	University degree	121	82.10	12.923	

Mother's job	Housewife	309	80.77	14.135	0.133
	Employed	61	83.66	10.936	

Father's job	Unemployed	10	79.06	13.344	0.774
	Worker	104	81.04	13.943	
	Employee	38	78.95	15.441	
	Freelance	191	81.90	13.273	
	Other	27	81.48	13.702	

School level	7th	108	81.68	13.357	0.879
	8th	147	81.31	14.518	
	9th	115	80.76	12.988	

Educational status	Bad	90	79.97	13.848	0.58
	Average	211	81.56	13.410	
	Good	69	81.97	14.402	

The child's underlying condition	Negative	350	80.96	13.832	0.032
	Positive	20	86.25	9.851	

Contracting COVID-19	Negative	19	79.93	13.353	0.664
	Positive	351	81.32	13.721	

Total	Min: 28.13	370	81.25	13.688	
	Max: 100.00				

**Table 3:** The performance of first-year high school students in the prevention of COVID-19.

		*N*	Mean	SD	*p* value
Age	14 >	101	62.16	25.685	0.097
	14 ≥	269	66.63	21.982	

Gender	Boy	184	64.31	23.872	0.366
	Girl	186	66.49	22.326	

Mothers' education	Diploma or less	237	64.93	23.023	0.601
	University degree	133	66.25	23.307	

Fathers' education	Diploma or less	249	66.18	22.671	0.368
	University degree	121	63.82	23.983	

Mother's job	Housewife	309	64.90	23.733	0.349
	Employed	61	67.94	19.573	

Father's job	Unemployed	10	68.89	23.307	0.276
	Worker	104	63.14	21.413	
	Employee	38	59.65	29.388	
	Freelance	191	67.25	21.831	
	Other	27	67.90	27.448	

School level	7th	108	62.55	25.717	0.253
	8th	147	65.76	20.949	
	9th	115	67.63	23.062	

Educational status	Bad	90	65.56	26.286	0.406
	Average	211	64.30	21.651	
	Good	69	68.60	23.021	

The child's underlying condition	Negative	350	64.32	22.775	< 0.001
	Positive	20	84.44	20.834	

Contracting COVID-19	Negative	19	74.27	21.293	0.078
	Positive	351	64.93	23.127	

Total	Min: 11.11	370	65.40	23.102	
	Max: 100.00				

**Table 4:** The correlation between knowledge, attitude, and performance of first-year high school students in the prevention of COVID-19.

		Age	Knowledge	Attitude	Performance
Knowledge	Correlation coefficient	0.162^∗∗^	1		
	Statistical significance	0.002			
	*N*	370	370		

Attitude	Correlation coefficient	−0.014	0.395^∗∗^	1	
	Statistical significance	0.786	0.000		
	*N*	370	370	370	

Performance	Correlation coefficient	0.088	0.385^∗∗^	0.153^∗∗^	1
	Statistical significance	0.091	0.000	0.003	
	N	370	370	370	370

^∗∗^Correlation is significant at the 0.01 level (2-tailed).

## Data Availability

Data are available from the first author upon request.

## References

[1] Wang H., Paulson K. R., Pease S. A. (2022). Estimating Excess Mortality due to the COVID-19 Pandemic: A Systematic Analysis of COVID-19-Related Mortality, 2020-21. *The Lancet*.

[2] Chams N., Chams S., Badran R. (2020). COVID-19: A Multidisciplinary Review. *Frontiers in Public Health*.

[3] Silva Andrade B., Siqueira S., de Assis Soares W. R. (2021). Long-COVID and Post-COVID Health Complications: An Up-to-Date Review on Clinical Conditions and Their Possible Molecular Mechanisms. *Viruses*.

[4] Sharma A., Ahmad Farouk I., Lal S. K. (2021). COVID-19: A Review on the Novel Coronavirus Disease Evolution, Transmission, Detection, Control and Prevention. *Viruses*.

[5] Sedgwick D., Hawdon J., Räsänen P., Koivula A. (2022). The Role of Collaboration in Complying With COVID-19 Health Protective Behaviors: A Cross-National Study. *Administration & Society*.

[6] Fegert J. M., Vitiello B., Plener P. L., Clemens V. (2020). Challenges and Burden of the Coronavirus 2019 (COVID-19) Pandemic for Child and Adolescent Mental Health: A Narrative Review to Highlight Clinical and Research Needs in the Acute Phase and the Long Return to Normality. *Child and Adolescent Psychiatry and Mental Health*.

[7] Wen F., Meng Y., Cao H. (2020). Knowledge, Attitudes, Practices of Primary and Middle School Students at the Outbreak of COVID-19 in Beijing: A Cross-Sectional Online Study. *MedRxiv*.

[8] Fallahi A., Mahdavifar N., Ghorbani A. (2022). Public Knowledge, Attitude and Practice Regarding Home Quarantine to Prevent COVID-19 in Sabzevar City, Iran. *Journal of Military Medicine*.

[9] Alshammary F., Siddiqui A. A., Amin J. (2021). Prevention Knowledge and Its Practice Towards COVID-19 Among General Population of Saudi Arabia: A Gender-Based Perspective. *Current Pharmaceutical Design*.

[10] Tajvar A., Aghamolaei T., Mohseni S. (2021). Knowledge, Performance, and Attitude Towards Mask Use to Prevent and Control COVID-19 Outbreak Among a Group of Iranian People: A Cross-Sectional Study. *Shiraz E-Medical Journal.*.

[11] Peng Y., Pei C., Zheng Y. (2020). A Cross-Sectional Survey of Knowledge, Attitude and Practice Associated with COVID-19 Among Undergraduate Students in China. *BMC Public Health*.

[12] Mohamed Ahmed Ayed M., abd Elaziem Mohamed A., Mohamed Mahmoud T., Mohammed AbdElaziz S. (2021). Effect of Educational Intervention on Secondary School Students’ Knowledge, Practices and Attitudes Regarding COVID-19. *Egyptian Journal of Health Care*.

[13] Majhi M. M., Meghachandra Singh M., Borle A. L., Rajiv A. (2021). Knowledge, Attitude and Practices Towards COVID-19 Among Undergraduate Students in a Medical College of Delhi. *Indian Journal of Public Health Research & Development*.

[14] Aynalem Y. A., Akalu T. Y., Gebresellassie Gebregiorgis B., Sharew N. T., Assefa H. K., Shiferaw W. S. (2021). Assessment of Undergraduate Student Knowledge, Attitude, and Practices Towards COVID-19 in Debre Berhan University, Ethiopia. *PLoS One*.

[15] Noreen K., Rubab Z.-E., Umar M., Rehman R., Baig M., Baig F. (2020). Knowledge, Attitudes, and Practices Against the Growing Threat of COVID-19 Among Medical Students of Pakistan. *PLoS One*.

[16] Azlan A. A., Hamzah M. R., Sern T. J., Ayub S. H., Mohamad E. (2020). Public Knowledge, Attitudes and Practices Towards COVID-19: A Cross-Sectional Study in Malaysia. *PLoS One*.

